# Prognostic value of the BAB index and a machine learning model integrating the BAB index for predicting mortality in acute ST-segment elevation

**DOI:** 10.3389/fnut.2025.1735916

**Published:** 2026-01-12

**Authors:** Haonan Xu, Tianshu Gu, Shuo Zhang, Shuang Zhao, Juan Xie, Jinhua Zhao, Gary Tse, Tong Liu, Kangyin Chen, Huaying Fu

**Affiliations:** 1 Tianjin Key Laboratory of Ionic-Molecular Function of Cardiovascular Disease Department of Cardiology, Tianjin Institute of Cardiology, The Second Hospital of Tianjin Medical University, Tianjin, China; 2 School of Public Health, Tianjin Medical University, Tianjin, China; 3 School of Nursing and Health Sciences, Hong Kong Metropolitan University, Hong Kong, China

**Keywords:** BAB index, biomarkers, machine learning, mortality, risk prediction, STEMI

## Abstract

**Background:**

The high mortality in ST-segment elevation myocardial infarction (STEMI) is associated not only with organ dysfunction and complications, but also with nutritional status. We aim to develop and validate a simple prognostic tool based on routinely serum biomarkers for predicting short- and long-term mortality in patients with STEMI, and to assess its contributing role in machine learning (ML) models.

**Methods:**

Observational multicenter data from the Tianjin Coronary Artery Disease (CAD) Database (2010–2021) were analyzed. The predictive abilities of biomarkers were identified via multivariable Cox regression. The BAB Index was calculated as Log_10_(NT-proBNP × ALT × BUN). Prognostic performance was evaluated by area under the curve (AUC) and compared with the CAMI-STEMI score. Validation included Cox regression, restricted cubic spline analysis (RCS), Kaplan–Meier survival, and subgroup analyses. ML models incorporating the BAB Index were constructed to verify the contributing roles of the BAB index in predicting 1-month and 1-year mortality.

**Results:**

Among 8,002 STEMI patients, BAB Index showed strong discriminatory power for 1-month (AUC = 0.804) and 1-year mortality (AUC = 0.794), comparable to the CAMI-STEMI score (*P* = 0.641). Higher BAB Index were independently associated with increased mortality (*P* < 0.001). RCS revealed a linear relationship, and Kaplan–Meier analysis confirmed worse survival with higher BAB Index (*P* < 0.001). Subgroup analyses demonstrated consistent findings. The XGBoost model achieved the highest performance for both 1-month (AUC 0.873) and 1-year mortality (AUC: 0.871), with BAB Index ranked among the top predictive features.

**Conclusions:**

BAB Index is a simple, effective tool for predicting short- and long-term mortality in STEMI. BAB index maintains a leading position among interpretable ML models.

## Introduction

ST-segment elevation myocardial infarction (STEMI) is a life-threatening cardiovascular condition associated with substantial short- and long-term morbidity and mortality worldwide (1, 2). Accurate forecasting of short- and long-term mortality risk remains a clinical challenge, particularly in identifying subtle but significant laboratory indicators that may not be captured by conventional scoring systems (3, 4). Therefore, early identification and precise clinical risk stratification remain critical to reducing cardiovascular complications and improving outcomes in intensive care settings.

The 2023 European Society of Cardiology (ESC) guidelines recommend the Global Registry of Acute Coronary Events (GRACE) risk score (Class IIa recommendation) as a preferred tool for assessing in-hospital mortality risk, citing its high diagnostic accuracy (5). In the Chinese population, the CAMI-STEMI score has also demonstrated reliable performance in predicting mortality. While these models are effective, individual risk assessment could be further enhanced through the integration of readily accessible biomarkers. Biomarkers offer a rapid, simple, and cost-effective means of stratifying risk in the acute care setting.

In this study, we primarily aim to explore the relationship between routine indicators, reflecting systemic organ function and nutritional status, with the mortality risk of STEMI, and subsequently develop a comprehensive integrated index. Furthermore, we would establish machine learning-based mortality prediction models incorporating the novel Index to further validate the contributing role of the novel index besides common risk factors.

## Materials and methods

### Data source

This study utilized data from the multicenter Coronary Artery Disease (CAD) specialized database housed within the Tianjin Health and Medical Big Data Super Platform (hereinafter referred to as “the Platform”). The Platform is operated by Tianjin Health and Medical Big Data Co., Ltd., an authorized institution responsible for comprehensive data management, including data collection, curation, governance, and application.

The Platform integrates clinical diagnostic and treatment data from 43 tertiary and 39 secondary hospitals across Tianjin, China, along with relevant public health information. Prior to inclusion in the research database, all data were standardized and de-identified, ensuring both interoperability and data security for academic use.

Specifically, the CAD-specific database comprises records of patients who were hospitalized between January 1, 2010, and March 31, 2024, with discharge diagnoses indicative of coronary artery disease. For each case, a wide range of healthcare information was collected, including demographic characteristics, clinical diagnoses, medication and non-medication prescriptions, diagnostic and laboratory test results, surgical interventions, healthcare expenditures, community pharmacy data, routine health check-up records, and mortality data sourced from public health systems.

### Study design and population

This study was conducted in accordance with the principles of the Declaration of Helsinki and was approved by the Ethics Committee of the Second Hospital of Tianjin Medical University (IRB number: KY2023052-01). It was designed as a multicenter, retrospective cohort study involving patients diagnosed with STEMI who were hospitalized between January 2010 and December 2021. STEMI diagnoses were confirmed by the attending physicians at discharge based on standard clinical, electrocardiographic, and biochemical criteria.

The study was conducted in three phases. In the first phase, we developed and internally validated a novel prognostic tool derived from routinely available clinical parameters. In the second phase, patients were categorized into tertiles based on the novel biomarker-based index values to assess its prognostic value for all-cause mortality at one month and one year. In the third part, we established machine learning-based mortality prediction models incorporating the novel index to further establish the contributing role of this novel biomarker-based index.

Inclusion criteria were as follows: (1) age >18 years; (2) a confirmed diagnosis of STEMI; and (3) availability of complete baseline and follow-up data. Patients were excluded if they had any of the following prior to index MI: (1) other pre-existing significant cardiac conditions (e.g., heart failure, significant valvular heart disease, or cardiomyopathy); (2) malignancies or severe systemic illnesses; (3) severe hepatic dysfunction or a known history of chronic liver disease; (4) preexisting chronic kidney disease [defined as an estimated glomerular filtration rate (eGFR) < 60 mL/min/1.73 m^2^]; or (5) planned non-emergency surgical procedures that could confound the short- or long-term outcomes.

Eligible patients were randomly assigned to a training cohort and an internal validation cohort at a 7:3 ratio. An external validation cohort was independently established using consecutive patients hospitalized with STEMI at the Second Hospital of Tianjin Medical University between January 2022 and December 2023.

Severe heart disease was defined as the presence of heart failure, significant valvular disease, or cardiomyopathy. Cardiac insufficiency was identified based on NT-proBNP thresholds, consistent with the 2021 ESC guidelines for heart failure: >450 pg/mL for patients aged < 55 years, >900 pg/mL for those aged 55–75 years, and >1,800 pg/mL for those aged >75 years.

### Endpoints

The primary endpoint of this study was all-cause mortality occurring within one month after hospital admission for STEMI, while the secondary endpoint was all-cause mortality within one year following admission.

### Data extraction

Baseline and follow-up data were extracted from the coronary artery disease database and included demographic and clinical characteristics such as age, sex, and comorbidities including hypertension, diabetes mellitus, and hyperlipidemia. Because peak values may be unavailable at decision-making time, laboratory results were used the first test results on admission before reperfusion therapy comprised hematological parameters from the complete blood count, hepatic function tests including ALT and aspartate aminotransferase (AST), renal function, cardiac biomarkers such as NT-proBNP, and lipid profiles including low-density lipoprotein cholesterol (LDL-C), high-density lipoprotein cholesterol (HDL-C), and triglycerides.

Clinical assessment at admission was performed using the Killip classification to evaluate the severity of heart failure. In-hospital medication use was recorded, covering major cardiovascular drugs such as angiotensin-converting enzyme inhibitors (ACEIs), angiotensin II receptor blockers (ARBs), angiotensin receptor–neprilysin inhibitors (ARNIs), β-blockers, calcium channel blockers (CCBs), diuretics, and antiplatelet agents.

Outcome data on all-cause mortality at one month and one year post-admission were collected during follow-up to evaluate the primary and secondary endpoints of the study.

## Statistical analysis

Continuous variables were presented as the mean ± standard deviation or the median with interquartile range (25th−75th percentiles), and compared using either the *t*-test or Mann–Whitney *U*-test, as appropriate. Categorical variables were expressed as frequencies (percentages) and compared using the Chi-squared test.

For datasets with less than 30% missing values, imputation was performed. For continuous variables, a differential strategy was applied depending on the results of the normality test: variables with *P* > 0.05 were imputed using the arithmetic mean, while those with *P* ≤ 0.05 were imputed using the median. Categorical variables were imputed using the mode (Supplementary Table 1).

In the first part, participants were randomly assigned to a training cohort and an internal validation cohort in a 7:3 ratio. Univariable Cox regression analyses were conducted for each clinical variable, and those with a *P*-value < 0.05 were included in a multivariable Cox regression model to evaluate the predictive value of laboratory results for one-month all-cause mortality. A simplified formula was derived from the regression coefficients and hazard ratios based significant biomarkers in the training cohort to establish the novel index (named BAB index).

Based on the regression results, receiver operating characteristic (ROC) curves were generated to assess the performance of the BAB index in predicting the primary endpoint. The optimal cut-off value was determined using Youden's index. The area under the curve (AUC), with corresponding 95% confidence intervals (CIs) and *P*-values, was reported. The predictive performance of the BAB index was compared with the CAMI-STEMI score using ROC analysis, net reclassification improvement (NRI), integrated discrimination improvement (IDI). The differences in AUC were evaluated using the DeLong test. Kaplan–Meier survival analysis was performed to compare outcomes between patients stratified by the BAB index cut-off, with significance assessed using the log-rank test. The predictive value of the BAB index was further validated in both the internal and external validation cohorts through ROC analysis and Kaplan–Meier curves.

In the second part, the population was stratified into tertiles based on BAB index scores. Three multivariable Cox regression models, adjusted for different sets of covariates, were used to assess the association between the BAB index and short- and long-term mortality. A restricted cubic spline (RCS) model was applied to explore potential non-linear relationships between the BAB index and the risk of one-month and one-year mortality. Subgroup analyses and interaction tests were conducted to evaluate the consistency of predictive performance across clinical subgroups, including interactions between culprit vessels and the BAB index. The association between the BAB index and secondary endpoints was assessed using similar statistical approaches.

In the third part, machine learning techniques were employed to develop mortality prediction models and to examine the contribution of the BAB index. Key predictors were selected using the LASSO regression algorithm, and multicollinearity was evaluated using the variance inflation factor (VIF). Five machine learning algorithms—XGBoost, Logistic Regression, LightGBM, AdaBoost, and GBDT—were used to build prediction models for one-month and one-year mortality. Model performance was evaluated through five repetitions of 5-fold cross-validation, and assessed using AUC, accuracy, and *F*1-score. For the top-performing model, SHapley Additive exPlanations (SHAP) were used to interpret feature importance, and partial dependence plots (PDPs) were generated to visualize the relationship between key predictors and outcomes.

All statistical analyses were performed using R version 4.4 and Python. A two-tailed *P*-value < 0.05 was considered statistically significant.

## Results

### Baseline characteristics

A total of 8,322 STEMI patients were included in the study after applying the inclusion and exclusion criteria. Among them, 8,002 patients (75.0% male, median age 63.0 years) were included in the primary analysis and randomly divided into a training cohort (*n* = 5,601) and an internal validation cohort (*n* = 2,401) at a 7:3 ratio. The one-month and one-year all-cause mortality rates in the overall population were 3.6% (290 events) and 5.7% (456 events), respectively. Regardless of whether patients died at one month or one year, they shared a common profile of advanced age, a higher proportion of females, higher KILLIP class, and a greater burden of comorbidities (Table 1 and Supplementary Table 2). No significant differences in baseline characteristics were observed between the training and validation cohorts. The one-month mortality rates were 3.7% and 3.5% in the training and internal validation cohorts, respectively. Baseline characteristics of the training cohort and internal validation cohort are summarized in Supplementary Table 3. Additionally, an external validation cohort consisting of 320 patients (20.3% male, median age 66.5 years) was included, with detailed characteristics presented in Supplementary Table 4.

**Table 1:** Baseline characteristics of the cohort on one-month mortality.

**Variable**	**Total (8,002)**	**Survivor (*n* = 7,712)**	**Death (*n* = 290)**	***P***
Age, *y*	63.0 (54.0,71.0)	62.0 (54.0,70.0)	75.0 (67.0,81.0)	< 0.001
Male, *n* (%)	6,000 (75.0)	5,815 (75.4)	185 (63.8)	< 0.001
Hypertension, *n* (%)	2,790 (34.9)	2,651 (34.4)	139 (47.9)	< 0.001
Diabetes, *n* (%)	2,207 (27.6)	2,107 (27.3)	100 (34.5)	< 0.001
Hyperlipidemia, *n* (%)	3,023 (37.8)	2,890 (37.5)	133 (45.9)	0.004
**KILLIP**, ***n*** **(%)**				
I	5,070 (63.4)	5,006 (64.9)	64 (22.1)	< 0.001
II	2,050 (25.6)	2,004 (26.0)	46 (15.9)	
III	482 (6.0)	403 (5.2)	79 (27.2)	
IV	400 (5.0)	299 (3.9)	101 (34.8)	
CCI	0 (0.1)	0 (0.1)	1 (0.2)	< 0.001
HFRS	2.9 (2.9, 5.5)	3.0 (2.9, 5.5)	2.9 (2.9, 5.9)	0.795
PCI, *n* (%)	5,071 (63.4)	4,904 (63.6)	167 (57.6)	0.037
ALT, U/L	32.4 (20.0, 52.0)	32.0 (20.0, 52.0)	37.0 (21.0, 80.0)	< 0.001
AST, U/L	103.0 (60.0, 167.4)	103.0 (59.6, 167.0)	103.0 (79.0, 187.3)	0.086
Creatine, μmol/L	71.7 (61.0, 85.0)	71.0 (60.8, 84.0)	91.2 (72.1, 120.6)	< 0.001
BUN, mmol/L	5.5 (4.4, 6.8)	5.4 (4.4, 6.7)	7.8 (5.7, 11.1)	< 0.001
NT-proBNP, ng/L	634.5 (184.0, 1,844.75)	606.5 (175.4, 1,710.0)	4,512.0 (1,245.3, 10,043.5)	< 0.001
LDL-C, mmol/L	3.1 (2.5, 3.6)	3.1 (2.5, 3.6)	3.1 (2.4, 3.5)	0.127
HDL-C, mmol/L	1.1 (0.9, 1.2)	1.1 (0.9, 1.2)	1.1 (0.9, 1.2)	0.632
Triglyceride, mmol/L	1.4 (1.0,2.1)	1.4 (1.1, 2.1)	1.4 (1.0, 1.6)	< 0.001
Hemoglobin, g/L	138.0 (126.0,149.0)	138.0 (127.0, 149.0)	132.0 (118.3, 141.0)	< 0.001
Platelet, 10^9^/L	220.0 (184.0,262.0)	220.0 (185.0, 263.0)	207.5 (166.3, 247.5)	< 0.001
ACEI/ARB/ARNI, *n* (%)	5,350 (66.9)	5,259 (68.2)	91 (31.4)	< 0.001
β-blocker, *n* (%)	5,384 (67.3)	5,269 (68.3)	115 (39.7)	< 0.001
CCB, *n* (%)	606 (7.6)	589 (7.6)	17 (5.9)	0.262
Diuretic, *n* (%)	3,025 (37.8)	2,901 (37.6)	124 (42.8)	< 0.001
Antiplatelet, *n* (%)	7,189 (89.8)	6,967 (90.3)	222 (76.6)	< 0.001

Continuous variables are expressed a median (25th, 75th); categorical variables are expressed as frequencies (percentages). CCI, Charlson comorbidity index; HFRS, hospital frailty risk score; PCI, percutaneous coronary intervention; ALT, alanine aminotransferase; AST, aspartate aminotransferase; BUN, blood urea nitrogen; NT-proBNP, N-terminal pro brain natriuretic peptide; LDL-C, low density lipoprotein cholesterol; HDL-C, high density lipoprotein cholesterol; ACEI, angiotensin-converting enzyme inhibitor; ARB, angiotensin receptor antagonists; ARNI, angiotensin receptor-neprilysin inhibitor; CCB, calcium channel blockers.

### NT-proBNP, ALT, and BUN are independent prognostic factors for 1-month mortality

As shown in Supplementary Table 5, univariable Cox regression analysis identified multiple factors significantly associated with one-month all-cause mortality (*P* < 0.05). These included demographic characteristics (older age, male), clinical history (hypertension, diabetes mellitus, and hyperlipidemia), treatment status [receipt of percutaneous coronary intervention (PCI)], and routine laboratory parameters such as ALT, AST, LDL-C, creatinine, NT-proBNP, HDL-C, triglycerides, hemoglobin, and platelet count.

Medication use, including CCBs, ACEIs, antiplatelet agents, and β-blockers, was also significantly associated with the outcome.

In the multivariable Cox regression analysis, several variables remained independently associated with increased risk of one-month mortality. These included older age, receipt of PCI, a history of diabetes mellitus, elevated levels of ALT, BUN, and NT-proBNP, as well as the use of ACEIs and β-blockers (all *P* < 0.05).

### Development of the BAB index

Finally, among included biomarkers, ALT, BUN, and NT-proBNP showed significant predictive ability in one-month mortality. Although HRs were close to 1.0 per unit increase, the wide measurement range of these biomarkers confers substantial prognostic gradient when scaled across their observed distributions. Therefore, based on the hazard ratios of ALT, BUN, and NT-proBNP identified in the multivariable Cox regression analysis, we developed a new composite index, referred to as the BAB index, using the following formula considering both clinical synergy and statistical parsimony:


$$\begin{array}{l} BAB\;index=Log_{10}(NT-proBNP\;(ng/L)\;\times \;ALT\;(U/L)\\ \times \;BUN(mmol/L)) \end{array}$$

Taking the base 10 logarithm of the product yielded an additive log-scale index that stabilized skewed distributions, reduced leverage from extreme values, and could be interpreted as the sum of standardized organ-stress signals.

**Figure 1:**
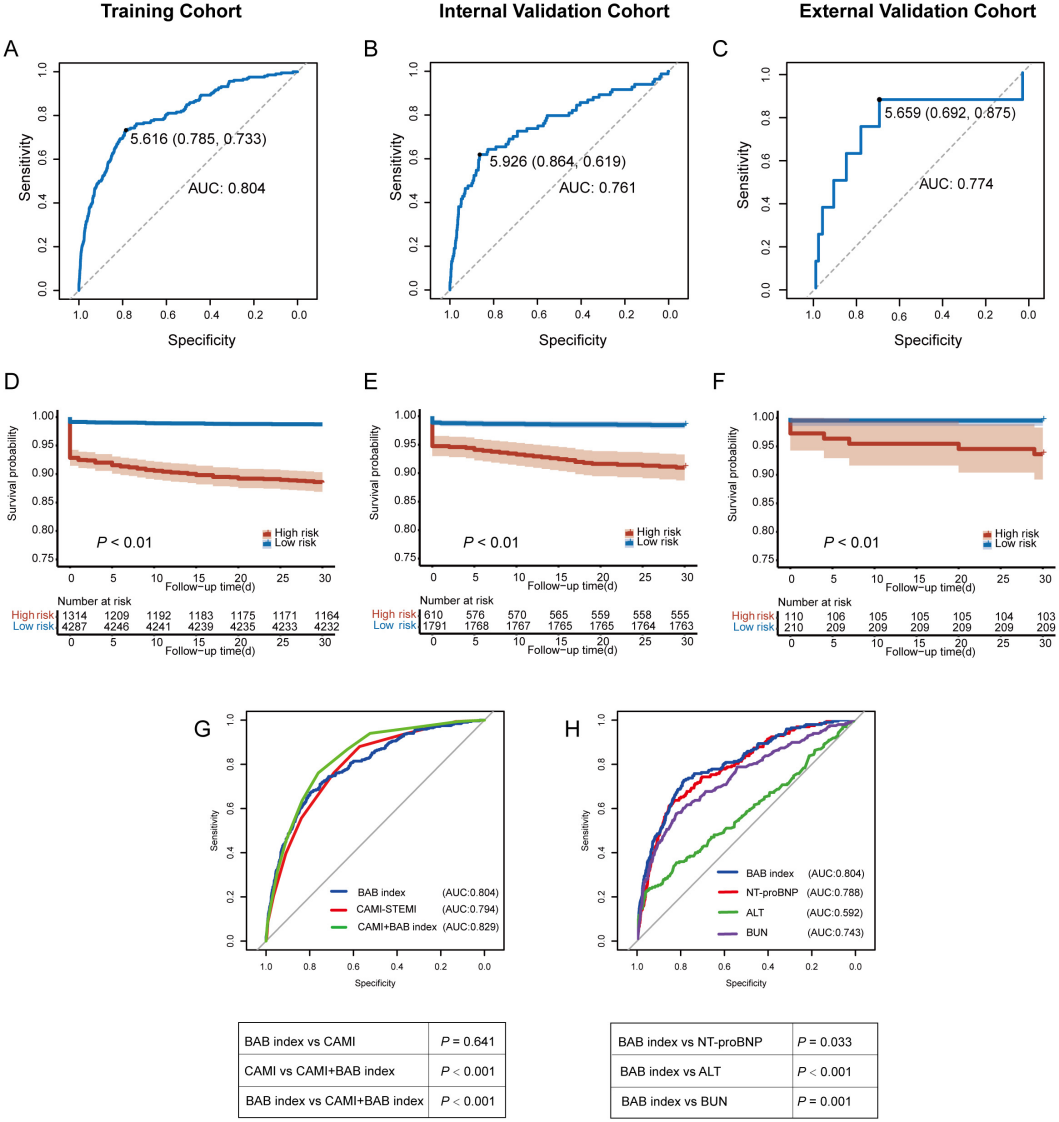
Results for one-month mortality. Receiver Operating Characteristic (ROC) curves and Kaplan–Meier curves for one-month all-cause mortality in the training cohort **(A, D)**, internal validation cohort **(B, E)**, and external validation cohort **(C, F)**. **(G)** Comparison of the predictive value of the BAB index and the CAMI-STEMI score for one-month all-cause mortality. **(H)** Comparison of the BAB index with NT-proBNP, ALT, and BUN, respectively. AUC, area under the curve; NT-proBNP, N-terminal pro B-type natriuretic peptide; ALT, alanine aminotransferase; BUN, blood urea nitrogen.

In the training cohort, Cox regression analysis revealed that the BAB index was independently associated with one-month all-cause mortality (HR: 2.951; 95% CI: 2.645–3.294; *P* < 0.001; Supplementary Table 6). The ROC curve for the BAB index showed good discriminative ability, with an AUC of 0.804, a sensitivity of 0.733, and a specificity of 0.785 (Figure 1A). The optimal cut-off value for predicting one-month mortality was 5.616, determined using Youden's index.

Compared with using NT-proBNP, ALT, or BUN individually, the BAB index demonstrated significantly superior predictive performance for short-term mortality (*P* = 0.033, *P* < 0.001, and *P* = 0.001, respectively; Figure 1H).

Using the cut-off value of 5.616, patients were stratified into low-risk (BAB index ≤ 5.616) and high-risk (BAB index > 5.616) groups. Kaplan–Meier survival analysis revealed that the high-risk group had significantly higher one-month all-cause mortality (*P* < 0.01; Figure 1D).

Furthermore, the BAB index was also an independent predictor of one-year all-cause mortality in the training cohort (HR: 2.756; 95% CI: 2.513–3.022; *P* < 0.001; Supplementary Table 6). The AUC for predicting one-year mortality was 0.794, with a sensitivity of 0.670 and a specificity of 0.799 (Supplementary Figure 1A). Consistently, the high-risk group showed significantly higher one-year all-cause mortality compared with the low-risk group (*P* < 0.01; Supplementary Figure 1D).

### Validation of the BAB index

We calculated the BAB index in both the internal and external validation cohorts using the formula derived from the training cohort. The BAB index was a significant predictor of one-month all-cause mortality in the internal cohort (HR: 2.601; 95% CI: 2.185–3.097; *P* < 0.001) and the external cohort (HR: 3.271; 95% CI: 1.595–6.708; *P* < 0.001) (Supplementary Table 6). The AUC was 0.761 (sensitivity: 0.619; specificity: 0.864) internally and 0.774 (sensitivity: 0.875; specificity: 0.692) externally (Figures 1B, C).

Using the cut-off value from the training cohort (BAB index ≤ 5.616 as low-risk and > 5.616 as high-risk), patients were stratified accordingly. Kaplan–Meier analysis revealed significantly higher one-month all-cause mortality in the high-risk groups across both validation cohorts (Figures 1E, F).

Similarly, the BAB index independently predicted one-year all-cause mortality in both cohorts. The hazard ratio was 2.570 (95% CI: 2.232–2.958; *P* < 0.001) in the internal cohort and 3.483 (95% CI: 1.828–6.637; *P* < 0.001) in the external cohort (Supplementary Table 6). The corresponding AUCs were 0.771 (sensitivity: 0.645; specificity: 0.814) internally and 0.805 (sensitivity: 0.900; specificity: 0.697) externally (Supplementary Figures 1B, C). Consistent with one-month results, high-risk groups had significantly higher one-year mortality rates in both cohorts (Supplementary Figures 1E, F).

We further assessed the BAB index's prognostic value for specific adverse events—intracerebral hemorrhage, ischemic stroke, and cardiac death. The BAB index was significantly associated with increased risks of these events at both one-month and one-year follow-ups among STEMI patients (Supplementary Figure 2).

Finally, long-term predictive validity was confirmed during the 5-year follow-up, where a high BAB index remained significantly associated with increased all-cause mortality (HR: 2.314; 95% CI: 2.178–2.459; *P* < 0.001; (Supplementary Figure 3).

### Comparison to the CAMI-STEMI score

To further evaluate the clinical utility of the BAB index, we compared its performance with the CAMI-STEMI score. The CAMI-STEMI score includes seven risk factors: female gender, heart rate ≥100 bpm, age ≥ 70 years, systolic blood pressure ≤ 115 mmHg, Killip class > I, cardiac arrest, and anterior wall infarction. ROC curve analysis was performed to assess the predictive ability for one-month mortality in the training cohort. The CAMI-STEMI score yielded an AUC of 0.794 (sensitivity: 0.881; specificity: 0.572), while the BAB index demonstrated comparable predictive performance (AUC: 0.804 vs. 0.794, *P* = 0.641; Figure 1G). Compared with the CAMI-STEMI score, the BAB index provided modest but statistically significant improvement in mortality risk prediction, with a NRI of 0.033 (95% CI, 0.005–0.071; *P* < 0.01) and an IDI of 0.017 (*P* = 0.026).

Although the BAB index showed slightly lower sensitivity than the CAMI-STEMI score, it exhibited higher specificity. Notably, when the BAB index was incorporated into the CAMI-STEMI score, the combined model improved the AUC to 0.829. Despite a decrease in sensitivity to 0.761, specificity increased to 0.761, suggesting that the combined model may enhance risk stratification by reducing false-positive rates (Figure 1G).

### The relationship between BAB index tertiles, baseline risk factors, and mortality

Patients were stratified into three groups according to the tertiles of the BAB index, revealing significant differences in baseline characteristics among these groups. As detailed in Supplementary Table 7, patients in the highest tertile were older, predominantly female, and exhibited higher prevalences of hypertension, diabetes, and severe Killip class. Additionally, this group had a lower rate of PCI, indicating a more complex clinical profile with potentially fewer opportunities for intervention.

Cox regression analysis demonstrated that higher BAB index tertiles were significantly associated with increased mortality at both one month (HR: 2.839; 95% CI: 2.588–3.115) and one year (HR: 2.696; 95% CI: 2.496–2.912). Multivariable-adjusted results are summarized in Table 2.

**Table 2:** Cox regression analysis for the correlation between the BAB index tertile and mortality in STEMI patients.

**Variables**	**Model 1**		**Model 2**		**Model 3**	
	**HR (95% CI)**	***P*** **-value**	**HR (95% CI)**	***P*** **-value**	**HR (95% CI)**	***P*** **-value**
**1-month mortality**						
Total	2.839 (2.588–3.115)	< 0.001	2.359 (2.124–2.620)	< 0.001	2.008 (1.804–2.234)	< 0.001
Tertile 1	Reference		Reference		Reference	
Tertile 2	1.908 (1.167–3.120)	0.01	1.521 (0.928–2.492)	0.096	1.667 (1.013–2.742)	0.044
Tertile 3	9.463 (6.209–14.41)	< 0.001	5.359 (3.471–8.274)	< 0.001	5.200 (3.321–8.141)	< 0.001
*P* for trend	< 0.001		< 0.001		< 0.001	
**1-year mortality**						
Total	2.696 (2.496–2.912)	< 0.001	2.215 (2.030–2.417)	< 0.001	1.937 (1.768–2.123)	< 0.001
Tertile 1	Reference		Reference		Reference	
Tertile 2	2.628 (1.752–3.944)	< 0.001	2.111 (1.404–3.172)	< 0.001	2.256 (1.497–3.402)	< 0.001
Tertile 3	11.222 (7.809–16.125)	< 0.001	6.529 (4.502–9.470)	< 0.001	6.094 (4.155–8.939)	< 0.001
*P* for trend	< 0.001		< 0.001		< 0.001	

Model 1: unadjusted. Model 2: adjusted for age, gender. Model 3: adjusted for age, gender, acceptance of PCI, hypertension, diabetes mellitus, hyperlipidemia, the use of ACEI/ARB/ARNI, β-blocker, CCB, diuretic, antiplatelet and anticoagulant. PCI, percutaneous coronary intervention; ACEI, angiotensin-converting enzyme inhibitor; ARB, angiotensin receptor antagonists; ARNI, angiotensin receptor-neprilysin inhibitor; CCB, calcium channel blockers. HR, hazard ratio; CI, confidence interval.

**Figure 2:**
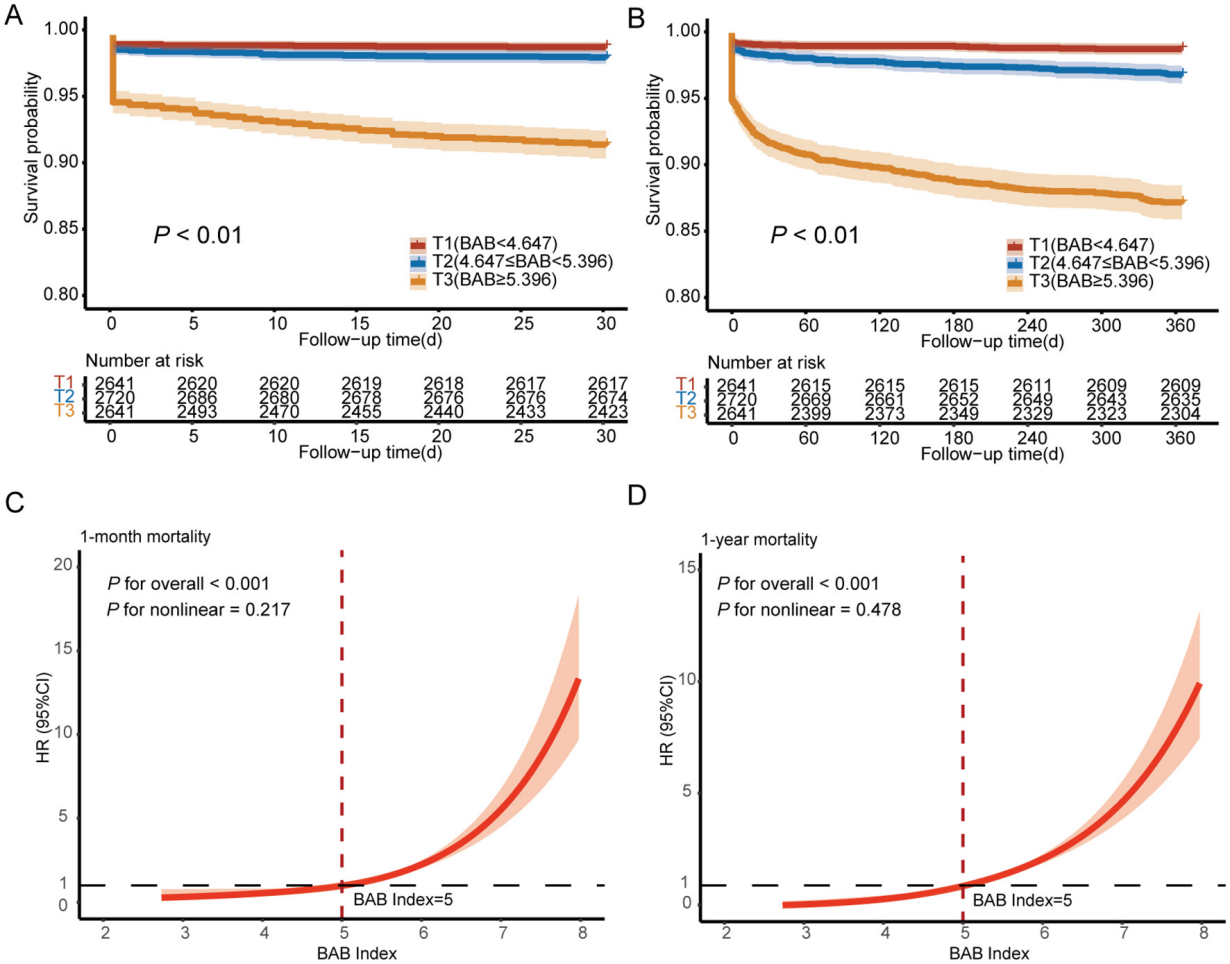
Results stratified by tertiles of the BAB index. Kaplan–Meier curves for one-month mortality risk **(A)** and one-year mortality risk **(B)** based on the BAB index in STEMI patients. **(C)** Restricted cubic spline curve of the BAB index in relation to one-month mortality. **(D)** Restricted cubic spline curve of the BAB index in relation to one-year mortality. STEMI, ST-segment elevation myocardial infarction.

Kaplan–Meier survival analysis further confirmed the prognostic value of BAB index tertiles, showing that patients in the highest tertile had significantly higher one-month and one-year mortality rates compared to those in lower tertiles (*P* < 0.01 by log-rank test; Figures 2A, B).

RCS analysis demonstrated a linear association between the BAB index and mortality risk at both one month (*P* for nonlinearity = 0.217; Figure 2C) and one year (*P* for nonlinearity = 0.478; Figure 2D), reinforcing the robustness of the linear relationship across the entire range of BAB index values.

### Subgroup and interaction analyses

**Figure 3:**
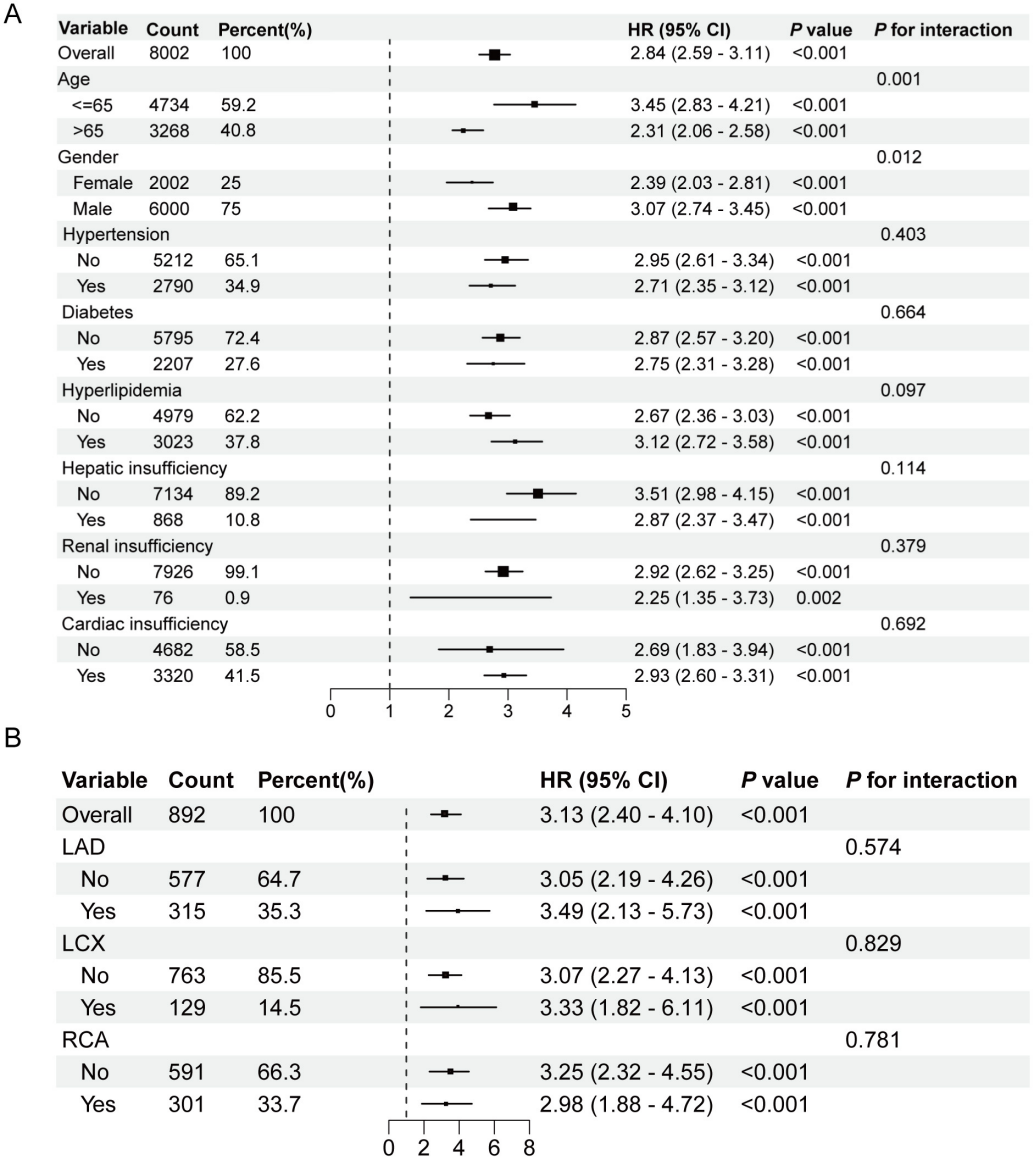
Subgroup analysis for one-month all-cause mortality. **(A)** Subgroup analysis by age, gender, hypertension, diabetes mellitus, hyperlipidemia, hepatic insufficiency, renal insufficiency, and cardiac insufficiency. **(B)** Subgroup analysis by culprit vessels. HR, hazard ratio; CI, confidence interval; PCI, percutaneous coronary intervention; LAD, left anterior descending artery; LCX, circumflex artery; RCA, right coronary artery.

Subgroup analyses were conducted based on age, gender, hypertension, diabetes mellitus, hyperlipidemia, hepatic insufficiency, renal insufficiency, and cardiac insufficiency (Figure 3A). The BAB index consistently and robustly predicted one-month mortality across all subgroups. Interaction analyses revealed that although younger and male patients had a higher overall risk of one-month mortality, the predictive performance of the BAB index remained stable across different age and gender groups. Moreover, the predictive value of the BAB index was not significantly affected by the presence or absence of other baseline comorbidities, including hypertension, diabetes mellitus, hyperlipidemia, hepatic insufficiency, renal insufficiency, or cardiac insufficiency (*P* for interaction > 0.05).

To further determine whether the BAB index predicts one-month mortality independently of the culprit vessel, we analyzed a subgroup of 892 patients with detailed coronary angiography records (Supplementary Table 8). The BAB index remained an independent predictor of one-month mortality across all culprit vessel categories, with no significant interaction between the BAB index and culprit vessel type (Figure 3B).

### Machine learning model construction and evaluation

**Figure 4:**
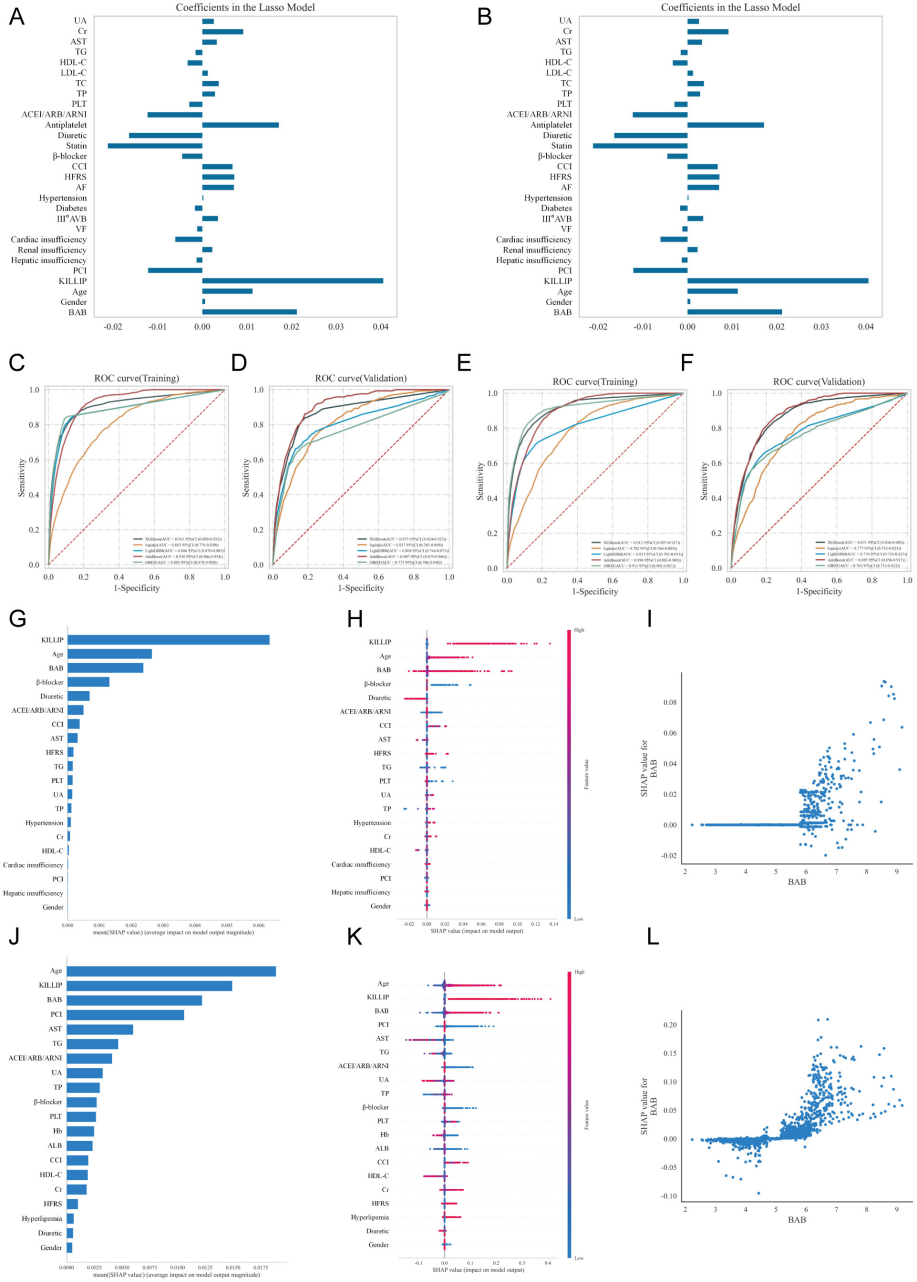
Prediction models using machine learning methods for one-month and one-year mortality, and SHAP values of all features in the XGBoost machine learning model. **(A, B**) Screening of key characteristics using the LASSO regression algorithm for one-month **(A)** and one-year **(B)** mortality risk. **(C, D)** ROC curve analysis of one-month mortality risk using different prediction models in the training **(C)** and validation **(D)** sets. **(E, F)** ROC curve analysis of one-year mortality risk using different prediction models in the training **(E)** and validation **(F)** sets. **(G)** Feature importance ranking for one-month mortality risk prediction. **(H)** SHAP summary plot for one-month mortality risk prediction. **(I)** Correlation analysis between the BAB index and SHAP values in one-month mortality risk prediction. **(J)** Feature importance ranking for one-year mortality risk prediction. **(K)** SHAP summary plot for one-year mortality risk prediction. **(L)** Correlation analysis between the BAB index and SHAP values in one-year mortality risk prediction. SHAP, SHapley Additive exPlanations; ROC, receiver operating characteristic.

To develop the 1-month mortality risk prediction model, we first identified 29 key features based on the Lasso regression. Multicollinearity diagnostics (Supplementary Table 9) revealed that four variables—statin, antiplatelet, TC, and LDL-C—exhibited significant collinearity issues (VIF > 5). These redundant variables were therefore excluded, leaving 25 features for subsequent model construction. The relative importance of these features is shown in Figure 4A. Similarly, for the 1-year mortality risk prediction model, 32 key features were initially selected by the Lasso regression model. The same four variables demonstrated collinearity (Supplementary Table 10) and were removed, resulting in 28 retained features (Figure 4B).

In terms of predictive performance, XGBoost outperformed other models for both 1-month mortality (AUC: 0.911, 95% CI: 0.889–0.932) and 1-year mortality (AUC: 0.912, 95% CI: 0.897–0.927) predictions (Figures 4C, E). On the validation set, XGBoost achieved an AUC of 0.873 (95% CI: 0.824–0.923), accuracy of 84.6% (95% CI: 82.4–86.8%), and *F*1-score of 0.276 (95% CI: 0.254–0.299) for the 1-month mortality prediction (Supplementary Table 11 and Figure 4D). To ensure robustness and prevent overfitting, we implemented 5-fold cross-validation combined with grid search for hyperparameter tuning. The model's strong performance was confirmed on an independent test set, which showed an improved AUC of 0.891 (95% CI: 0.859–0.923), accuracy of 84.2%, and *F*1-score of 0.225.

For the 1-year mortality prediction (Supplementary Table 12 and Figure 4F), XGBoost achieved an AUC of 0.871 (95% CI: 0.836–0.905), accuracy of 83.9% (95% CI: 81.3–86.6%), and *F*1-score of 0.343 (95% CI: 0.254–0.299) on the validation set, maintaining consistent performance on the test set (AUC: 0.883, 95% CI: 0.859–0.906; accuracy: 82.0%; *F*1-score: 0.303).

SHAP analysis further demonstrated that the BAB index was a consistently influential feature in both prediction models. For the 1-month mortality model (Figures 4G–I), BAB ranked third in feature importance, with higher values correlating positively with increased mortality risk. This relationship persisted in the 1-year mortality model (Figures 4K–L), where BAB maintained its third-place ranking and positive association with mortality risk. These findings underscore the clinical significance of the BAB index as a robust predictor for both short- and long-term mortality outcomes.

While complex machine learning models achieved higher AUCs, the BAB index offers interpretability and ease of manual calculation.

## Discussion

In this study, we conducted a multicenter observational study including 8,002 STEMI patients and developed a novel composite biomarker—the BAB index—derived from NT-proBNP, ALT, and BUN levels. The BAB index not only reflects the cardiac, hepatic, and renal function of patients after STEMI, but also indicates the potential nutritional status of the body under stress. Our results demonstrated that the BAB index is an independent predictor of all-cause mortality at both one month and one year following STEMI. Moreover, machine learning and SHAP analyses confirmed that the BAB index holds a prominent position among predictive features for both short- and long-term mortality.

### The need for improved risk stratification in STEMI patients

STEMI is associated with a high mortality rate. The pathophysiology of STEMI involves rupture or erosion of an atherosclerotic plaque, causing thrombotic occlusion of an epicardial coronary artery, which leads to myocardial infarction and myocardial stunning (6). Both large infarct size and myocardial stunning are well-established significant risk factors for all-cause mortality (7, 8). Furthermore, complications following STEMI—such as heart failure, cardiac arrhythmias, and bleeding—further increase mortality risk (5). Therefore, early risk assessment and stratification are critical (9).

In addition to recommended risk scores like the GRACE model, CAMI-STEMI score, numerous studies have sought to develop practical tools for mortality prediction based on clinical characteristics, cardiovascular imaging, and laboratory data (10–12). Among these approaches, the use of serum biomarkers offers a simpler, more accessible, and cost-effective method for mortality risk prediction in STEMI patients. Acute ischemic injury to the myocardium initiates neurohormonal activation, hemodynamic instability, and cardiac remodeling, all of which can induce changes in serum biomarkers reflecting cardiac, hepatic, and renal function (6, 13, 14).

### Association between the BAB index and STEMI mortality and underlying mechanisms

NT-proBNP is primarily secreted by left ventricular (LV) cardiomyocytes and serves as a marker of neurohormonal activation (15, 16). It is released in response to pressure or volume overload and LV remodeling (16). In STEMI patients, severe cardiac ischemia and microvascular injury stimulate NT-proBNP secretion from cardiomyocytes (15). Importantly, Granger et al. demonstrated that the prognostic value of NT-proBNP in this population is independent of heart failure, suggesting that systemic inflammation triggered by coronary plaque rupture may also regulate its expression (17). Elevated NT-proBNP levels have consistently been identified as independent predictors of major adverse cardiac events in STEMI patients (18, 19).

ALT elevation may result from liver hypoperfusion caused by left ventricular dysfunction, or from hepatic congestion due to right ventricular dysfunction. Additionally, ischemic injury to cardiomyocytes might contribute to increased ALT levels (20). ALT levels have been shown to correlate independently with infarct size and systemic inflammation (21). Since impaired hepatic function adversely affects patient outcomes, elevated serum ALT has been established as a risk factor for mortality in STEMI patients, independent of left ventricular ejection fraction, age, and sex (21, 22).

BUN reflects not only renal function but also protein metabolism and nutritional status, independent of eGFR (23). Elevated BUN levels in STEMI patients are associated with increased mortality risk (23). This association may be attributed to poor nutritional status, inappropriate neurohormonal activation, and kidney dysfunction caused by hypoperfusion (14, 23). Furthermore, increased BUN serves as a simple indicator of enhanced sodium and water reabsorption, which adds prognostic value for mortality prediction (23).

### Clinical implications and translational potential of the BAB index

The BAB index—comprising NT-proBNP, ALT, and BUN—can be readily calculated using standard laboratory tests obtained at admission. By integrating cardiac, hepatic, and renal biomarkers, the BAB index provides a multidimensional reflection of a patient's functional status and pathophysiological burden. In our study, this composite index demonstrated robust prognostic value for both short-term and long-term mortality in patients with STEMI.

Importantly, subgroup analyses confirmed that the prognostic utility of the BAB index remained consistent across patients with or without hepatic, renal, or cardiac insufficiency. Furthermore, no significant interaction was observed between the BAB index and culprit coronary artery location, indicating its broad applicability across diverse STEMI presentations. Though the BAB index showed interactions across different age and gender subgroups, it still demonstrated a significant positive predictive effect within each subgroup. Future studies with larger samples should explore age-specific thresholds to further optimize predictive performance across age groups. Of note, the predictive performance of the BAB index was comparable to that of the CAMI-STEMI score, a widely recognized multi-parameter risk model used in Chinese clinical practice. This finding suggests that the BAB index, despite being simpler and more accessible, may offer equivalent risk stratification utility.

**Figure 5:**
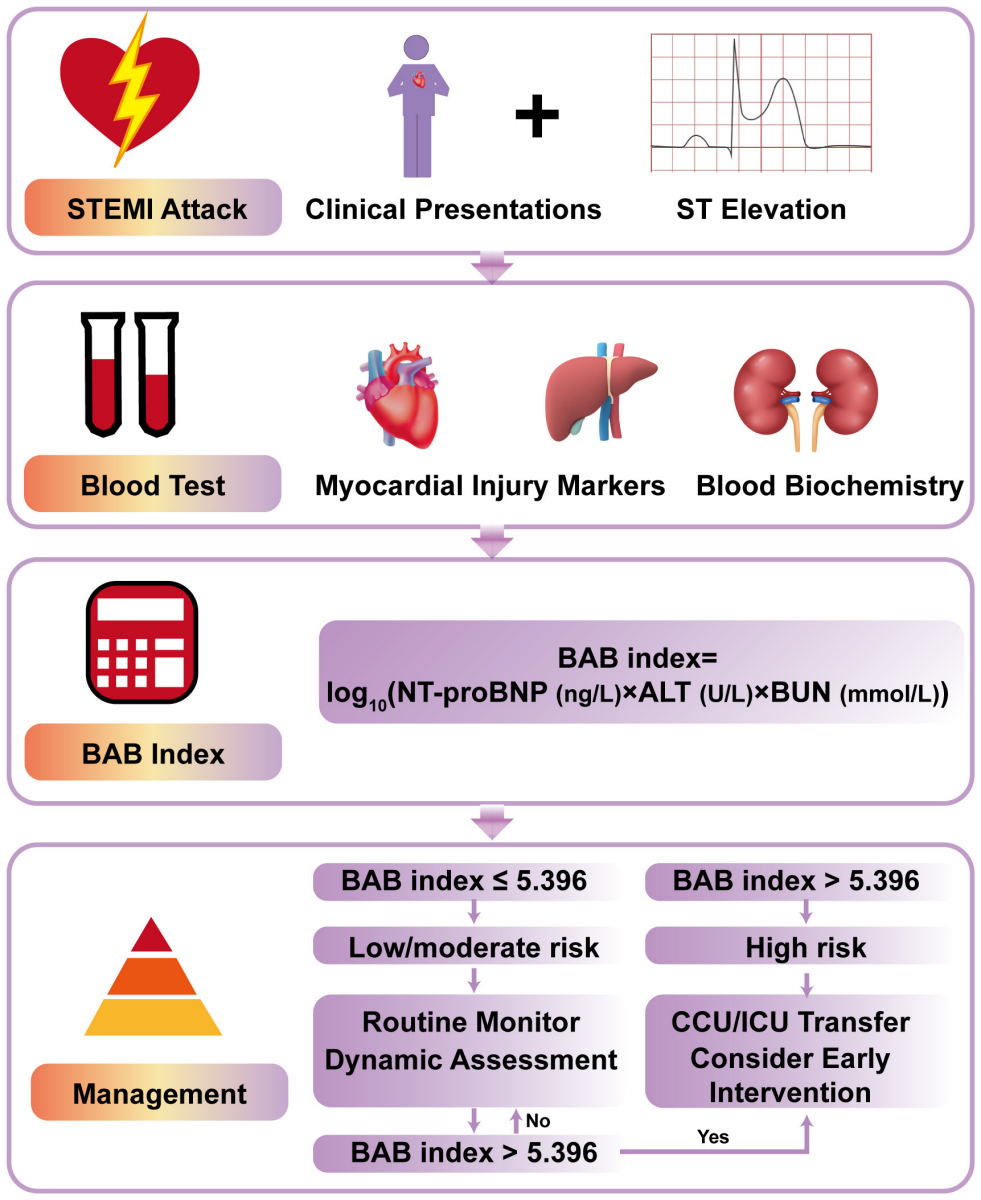
Flowchart for clinical risk stratification and management in patients with STEMI. The algorithm integrates laboratory biomarkers and clinical status to guide decisions regarding admission and treatment. STEMI, ST-segment elevation myocardial infarction; NT-proBNP, N-terminal pro B-type natriuretic peptide; ALT, alanine aminotransferase; BUN, blood urea nitrogen; CCU, cardiac care unit; ICU, intensive care unit.

To facilitate bedside application, we propose a clinical decision-making flowchart integrating the BAB index into current STEMI management pathways (Figure 5). For high-risk patients (top tertile of BAB index), early identification may prompt immediate intensive monitoring, aggressive medical therapy, and prompt transfer to a critical care unit, potentially reducing adverse outcomes. For low- to intermediate-risk patients, standard care and dynamic monitoring may suffice, with serial assessment of the BAB index potentially serving as an early indicator of treatment response or clinical deterioration.

This simplified, biomarker-driven approach may enable clinicians to implement timely, individualized, and resource-efficient treatment strategies in the acute management of STEMI.

### Limitations

This study was retrospective in design, and prospective validation is warranted. Because all components of the BAB index are serum biomarkers, variations in blood-sampling timing may influence their measured values. Although we used admission measurements for consistency, temporal changes in these biomarkers may also affect their prognostic performance. Future studies should therefore evaluate the prognostic value of dynamic biomarker trajectories. NT-proBNP appeared to contribute the majority of prognostic information, while the additional variables added modest incremental value. To avoid chronic elevations in NT-proBNP, ALT and BUN that may obscure acute prognostic signals, we excluded patients with a history of cardiac, hepatic or kidney disease; however, the predictive accuracy of the BAB index in such populations should be interpreted with caution. Future studies should validate the index in these populations. We used median/mean value for imputation, which may mask data distribution characteristics. Lastly, due to the absence of pre-hospital cardiac arrest data, we were unable to compare the BAB index directly with the GRACE risk score.

## Conclusions

**Figure 6:**
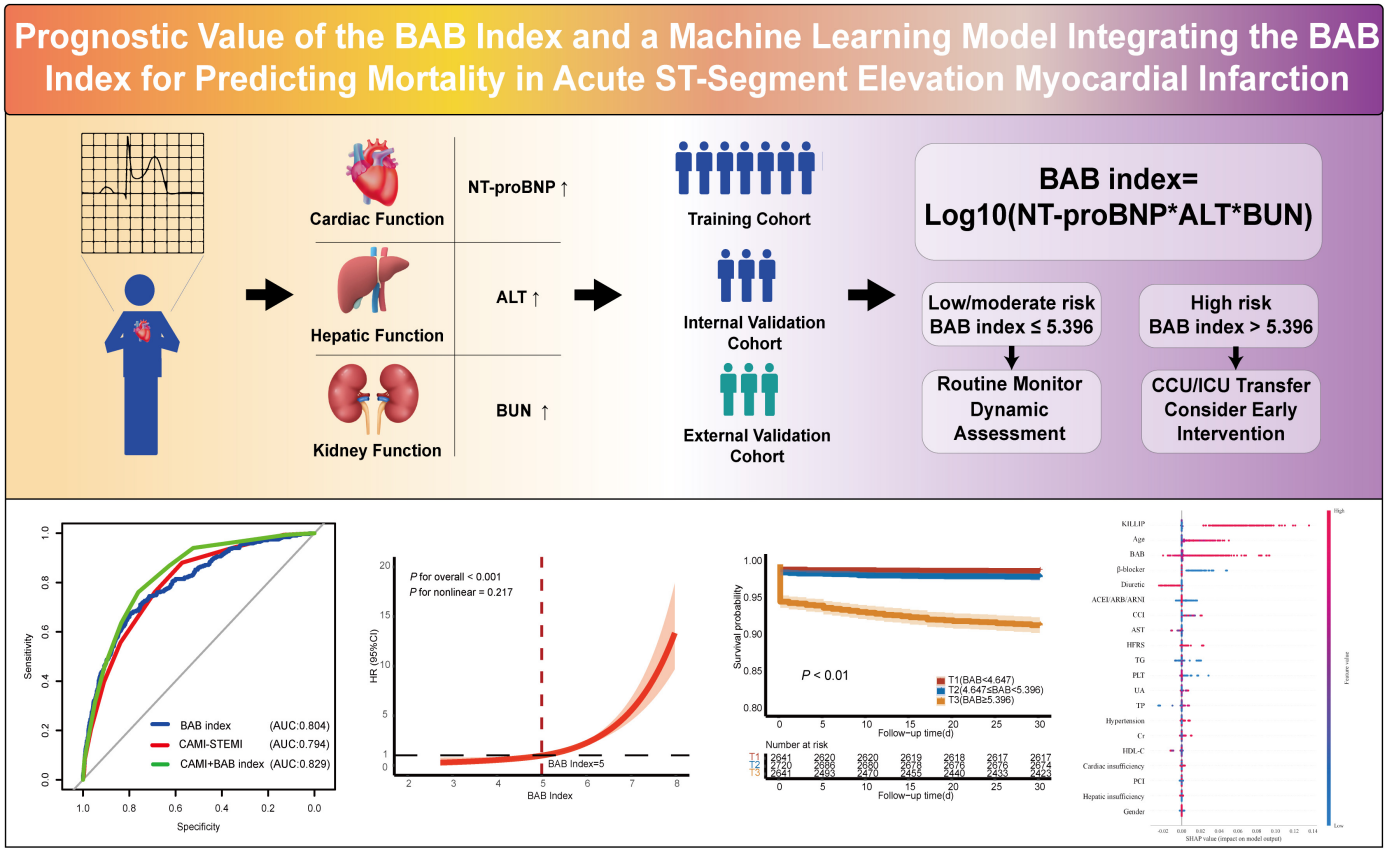
Central illustration: the BAB Index effectively predicts 1-month and 1-year mortality, matching or outperforming conventional scores, especially when integrated with machine learning models.

In this study, we developed a simple and non-invasive index—the BAB index. The BAB index was significantly associated with both short-term and long-term mortality in patients with STEMI. These findings suggest that the BAB index may serve as a practical and cost-effective tool, enabling timely identification of high-risk patients based solely on routine laboratory parameters (Figure 6).

## Data Availability

The raw data supporting the conclusions of this article will be made available by the authors, without undue reservation.
