# Supplementary material for: Prognostic value of the BAB index and a machine learning model integrating the BAB index for predicting mortality in acute ST-segment elevation
Source: Front Nutr. 2026 Jan 12;12:1735916. doi: 10.3389/fnut.2025.1735916 (PMC12833616; doi:10.3389/fnut.2025.1735916)
Supplement: Supplementary file 1 [file Table_1.docx]

Supplementary Material

# Supplementary Figures and Tables

## Supplementary Figures


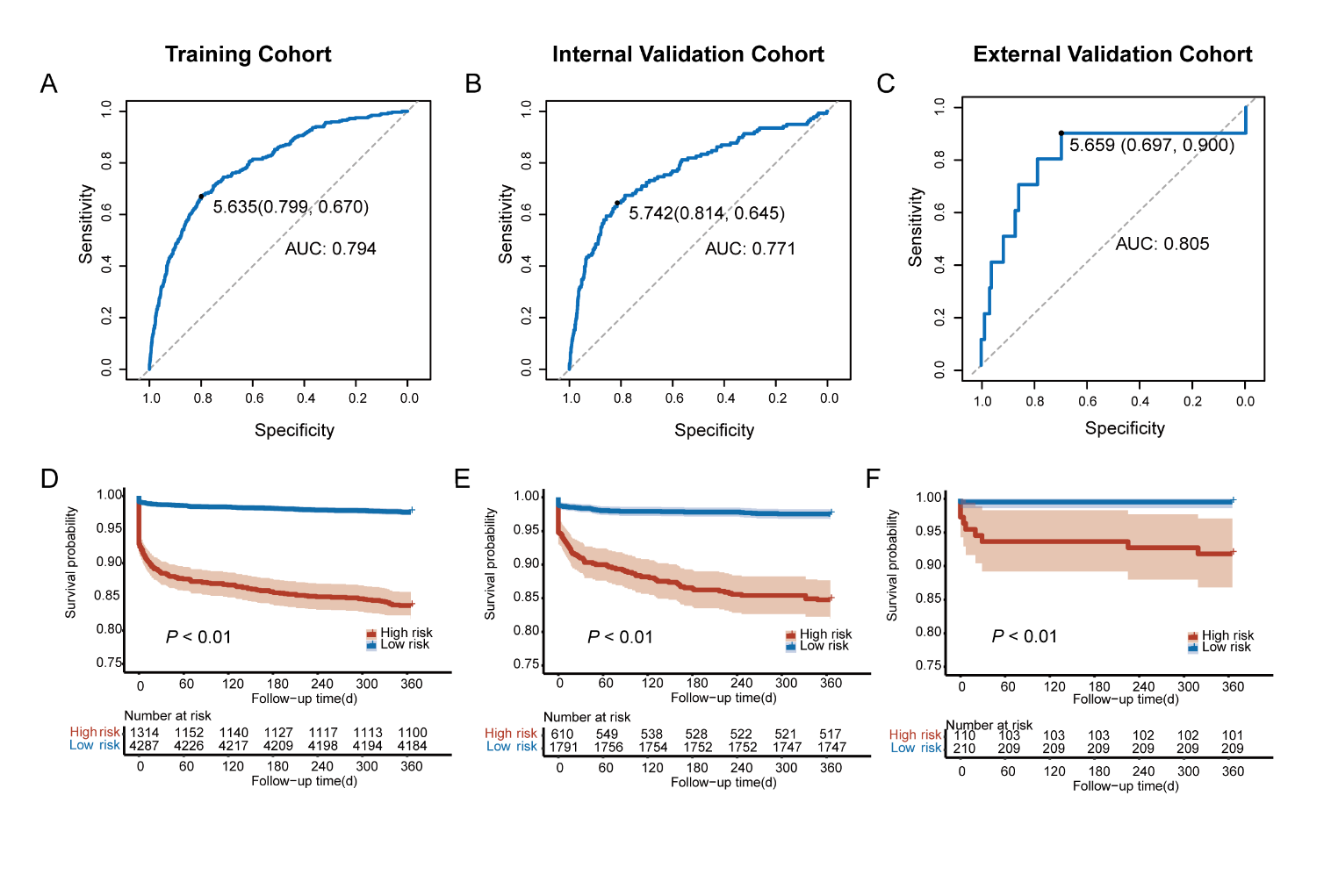


**Supplementary Figure 1. Results for one-year mortality.**

ROC curves and Kaplan–Meier curves for one-year all-cause mortality in the training cohort (A, D), internal validation cohort (B, E), and external validation cohort (C, F). Abbreviations: AUC, area under the curve.


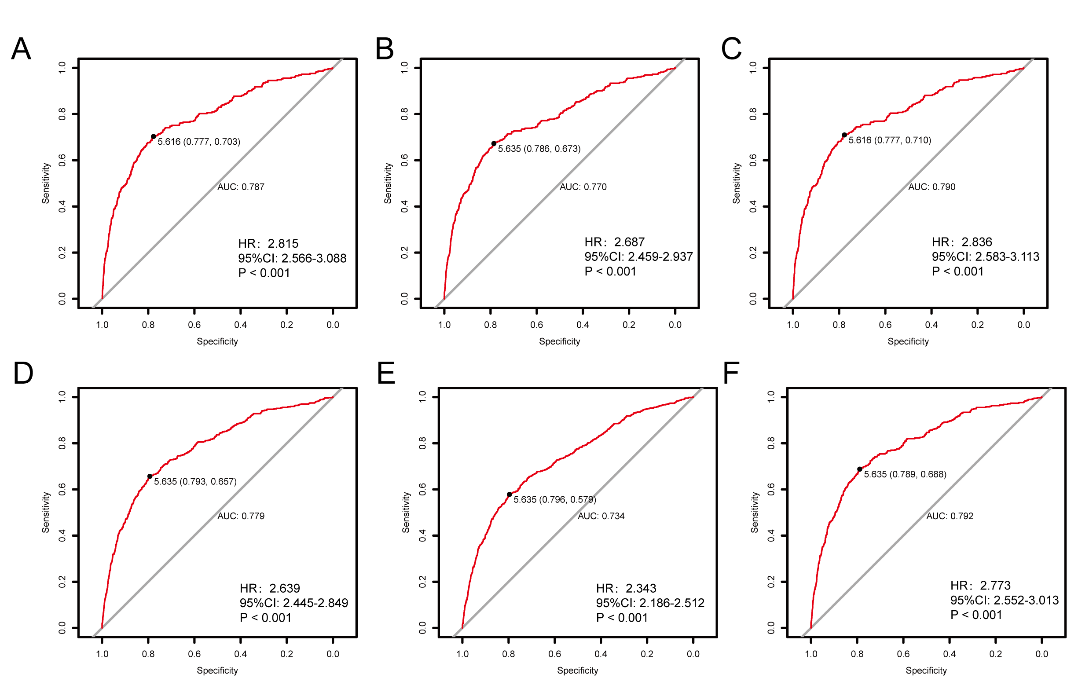


**Supplementary Figure 2. The BAB index was associated with an increased risk of 1-mon and 1-year intracerebral hemorrhage, ischemic stroke, and cardiac death of STEMI**

A: 1-month intracerebral hemorrhage; B: 1-month ischemic stroke; C: 1-month cardiac death; D: 1-year intracerebral hemorrhage; E: 1-year ischemic stroke; F: 1-year cardiac death. AUC: area under the curve**;** HR: hazards ratio; CI: confidence interval; STEMI: ST-segment elevation myocardial infarction.

**
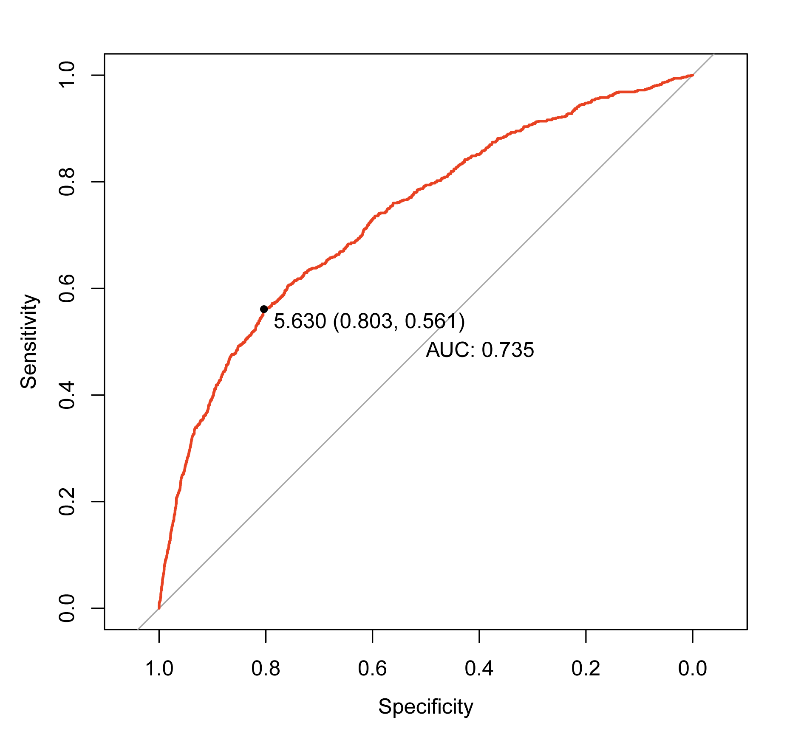
**

**Supplementary Figure 3. The high BAB index was associated with an increased risk of 5-year all-cause mortality.** AUC: area under the curve.

**Supplementary Table 1** Missing variables and imputation procedures

| **Variables** | **Missing Count** | **Missing Percentage(%)** | **Numerical distribution** | **Supplementary Missing Values** |
| --- | --- | --- | --- | --- |
| AST | 2087 | 26.0810 | Skewed distribution | Median imputation: 103.00 |
| PLT | 232 | 2.8993 | Skewed distribution | Median imputation: 220.00 |
| Hb | 223 | 2.7868 | Skewed distribution | Median imputation: 138.00 |
| LDL-C | 190 | 2.3744 | Skewed distribution | Median imputation: 3.05 |
| HDL-C | 189 | 2.3619 | Skewed distribution | Median imputation: 1.05 |
| TG | 121 | 1.5121 | Skewed distribution | Median imputation: 1.43 |
| TC | 120 | 1.4996 | Skewed distribution | Median imputation: 4.63 |
| UA | 71 | 0.8873 | Skewed distribution | Median imputation: 326.00 |
| ALB | 14 | 0.1750 | Skewed distribution | Median imputation: 39.00 |
| TP | 8 | 0.1000 | Skewed distribution | Median imputation: 63.40 |
| HFRS | 5 | 0.0625 | 0-1 distribution | Modal imputation: 0 |
| CCI | 5 | 0.0625 | Distribution of categorical variables | Modal imputation: 0 |
| ALT | 1 | 0.0125 | Skewed distribution | Median imputation: 32.40 |
| Cr | 1 | 0.0125 | Skewed distribution | Median imputation: 71.66 |

AST: aspartate aminotransferase; PLT: platelet; Hb: hemoglobin; BUN: blood urea nitrogen; LDL-C: Low density lipoprotein cholesterol; HDL-C: High density lipoprotein cholesterol; TG: triglyceride; TC: total cholesterol; UA: uric acid; ALB: albubin; TP: total protein; ALT: alanine aminotransferase; Cr: creatine; HFRS: Hospital Frailty Risk Score; CCI: Charlson Comorbidity Index.

**Supplementary Table 2** Baseline Characteristics of the cohort on one-year mortality.

| Variable | Total  (8002) | Survivor  (n=7546) | Death  (n=456) | *P* |
| --- | --- | --- | --- | --- |
| Age, y  Male, n (%)  Hypertension, n (%)  Diabetes, n (%)  Hyperlipidemia, n (%)  KILLIP, n (%)  Ⅰ  Ⅱ  Ⅲ  Ⅳ  CCI  HFRS  PCI, n (%)  ALT, U/L  AST, U/L  Creatine, umol/L  BUN, mmol/L  NT-proBNP, ng/L  LDL-C, mmol/L  HDL-C, mmol/L  Triglyceride, mmol/L  Hemoglobin, g/L  Platelet, 10^9/L  ACEI/ARB/ARNI, n (%)  β-blocker, n (%)  CCB, n (%)  Diuretic, n (%)  Antiplatelet, n (%) | 63.0(54.0,71.0)  6000(75.0)  2790(34.9)  2207(27.6)  3023(37.8)  5070(63.4)  2050(25.6)  482(6.0)  400(5.0)  0(0,1)  2.9(2.9,5.5)  5071(63.4)  32.4(20.0,52.0)  103.0(60.0,167.4)  71.66(61.0,85.0)  5.5(4.4,6.8)  634.5(184.0,1844.75)  3.1(2.5,3.6)  1.1(0.9,1.2)  1.4(1.0,2.1)  138.0(126.0,149.0)  220.0(184.0,262.0)  5350(66.9)  5384(67.3)  606(7.6)  3025(37.8)  7189(89.8) | 62.0(54.0,70.0)  5718(75.8)  2583(34.2)  2044(27.1)  2812(37.3)  4953(65.6)  1947(25.8)  372(4.9)  274(3.6)  0(0,1)  2.9(2.9,5.5)  4790(63.5)  32.0(20.1,51.9)  103.0(60.0,168.0)  71.0(60.8,84.0)  5.4(4.4,6.7)  585.9(170.2,1636.8)  3.1(2.5,3.6)  1.1(0.9,1.2)  1.4(1.1,2.1)  138.0(127.0,149.0)  220.0(185.0,262.9)  5162(68.4)  5160(68.4)  577(7.7)  2800(37.1)  6815(90.3) | 74.0(67.0,80.0)  282(61.8)  207(45.4)  163(35.8)  211(46.3)  117(25.7)  103(22.6)  110(24.1)  126(27.6)  0(0,2)  3.6(2.9,5.8)  281(61.6)  34.0(19.0,66.0)  103.0(64.0,157.6)  85.0(67.0,112.0)  7.4(5.7,10.0)  3995.5.0(1316.3,9087.3)  3.1(2.4,3.6)  1.1(0.9,1.3)  1.3(0.9,1.6)  130.0(117.0,140.0)  213.0(170.0,257.3)  188(41.2)  224(49.1)  29(6.4)  225(49.3)  374(82.0) | <0.001  <0.001  <0.001  <0.001  <0.001  <0.001  <0.001  0.538  0.425  0.022  0.886  <0.001  <0.001  <0.001  0.017  0.161  <0.001  <0.001  0.006  <0.001  <0.001  0.313  <0.001  <0.001 |

Continuous variables are expressed a median (25th, 75th); Categorical variables are expressed as frequencies (percentages). CCI: Charlson Comorbidity Index; HFRS: Hospital Frailty Risk Score; PCI: Percutaneous coronary intervention; ALT: alanine aminotransferase; AST: aspartate aminotransferase; BUN: blood urea nitrogen; NT-proBNP: N-terminal pro brain natriuretic peptide; LDL-C: Low density lipoprotein cholesterol; HDL-C: High density lipoprotein cholesterol; ACEI: angiotensin-converting enzyme inhibitor; ARB: Angiotensin receptor antagonists; ARNI: Angiotensin Receptor-Neprilysin Inhibitor; CCB: calcium channel blockers.

**Supplementary Table 3** Baseline Characteristics of the training cohort and internal validation cohort.

| Variable | Total  (8002) | Training Cohort  (n=5601) | Internal Validation  Cohort (n=2401) | *P* |
| --- | --- | --- | --- | --- |
| Age, y  Male, n (%)  Hypertension, n (%)  Diabetes, n (%)  Hyperlipidemia, n (%)  KILLIP, n (%)  Ⅰ  Ⅱ  Ⅲ  Ⅳ  CCI  HFRS  PCI, n (%)  ALT, U/L  AST, U/L  Creatine, umol/L  BUN, mmol/L  NT-proBNP, ng/L  LDL-C, mmol/L  HDL-C, mmol/L  Triglyceride, mmol/L  Hemoglobin, g/L  Platelet, 10^9/L  ACEI/ARB/ARNI, n (%)  β-blocker, n (%)  CCB, n (%)  Diuretic, n (%)  Antiplatelet, n (%)  1-month mortality, n (%)  1-year mortality, n (%) | 63.0(54.0,71.0)  6000(75.0)  2790(34.9)  2207(27.6)  3023(37.8)  5070(63.4)  2050(25.6)  482(6.0)  400(5.0)  0(0,1)  2.9(2.9,5.5)  5071(63.4)  32.4(20.0,52.0)  103.0(60.0,167.4)  71.66(61.0,85.0)  5.5(4.4,6.8)  634.5(184.0,1844.75)  3.1(2.5,3.6)  1.1(0.9,1.2)  1.4(1.0,2.1)  138.0(126.0,149.0)  220.0(184.0,262.0)  5350(66.9)  5384(67.3)  606(7.6)  3025(37.8)  7189(89.8)  290(3.6)  456(5.7) | 63.0(54.0,71.0)  4185(74.7)  1942(34.7)  1544(27.6)  2109(37.7)  3533(63.1)  1453(25.9)  338(6.0)  277(4.9)  0(0,1)  2.9(2.9,5.5)  3566(63.7)  32.0(20.0,51.0)  103.0(59.3,168.0)  71.5(61.0,85.0)  5.5(4.4,6.8)  615.9(174.5,1838.0)  3.1(2.5,3.6)  1.1(0.9,1.2)  1.4(1.0,2.1)  138.0(126.6,149.0)  220.0(184.0,263.0)  3766(67.2)  3789(67.6)  439(7.8)  2083(37.2)  5047(90.1)  206(3.7)  318(5.7) | 63.0(54.0,71.0)  1815(75.6)  848(35.3)  663(27.6)  914(38.1)  1537(64.0)  597(24.9)  144(6.0)  123(5.1)  0(0,1)  3.6(2.9,5.6)  1505(62.7)  34.0(21.0,55.0)  103.0(61.0,166.0)  72.0(61.0,84.8)  5.5(4.4,6.8)  674.0(209.0,1869.0)  3.1(2.5,3.6)  1.1(0.9,1.2)  1.4(1.0,2.0)  138.0(126.0,149.0)  220.0(184.0,261.0)  1584(66.0)  1595(66.4)  167(7.0)  942(39.2)  2142(89.2)  84(3.5)  138(5.8) | 0.545  0.424  0.596  0.987  0.746  0.776  0.106  0.082  0.416  0.198  0.204  0.789  0.574  0.988  0.736  0.516  0.112  0.404  0.514  0.282  0.299  0.186  0.089  0.240  0.743  0.943 |

Continuous variables are expressed a median (25th, 75th); Categorical variables are expressed as frequencies (percentages). CCI: Charlson Comorbidity Index; HFRS: Hospital Frailty Risk Score; PCI: Percutaneous coronary intervention; ALT: alanine aminotransferase; AST: aspartate aminotransferase; BUN: blood urea nitrogen; NT-proBNP: N-terminal pro brain natriuretic peptide; LDL-C: Low density lipoprotein cholesterol; HDL-C: High density lipoprotein cholesterol; ACEI: angiotensin-converting enzyme inhibitor; ARB: Angiotensin receptor antagonists; ARNI: Angiotensin Receptor-Neprilysin Inhibitor; CCB: calcium channel blockers.

**Supplementary Table 4.** Baseline Characteristic of External Validation Cohort.

| Variable | Total  (320) | Survival  (n=312) | Death  Cohort (n=8) | *P* |
| --- | --- | --- | --- | --- |
| Age,y  Male, n(%)  Hypertension, n(%)  Diabetes, n(%)  Hyperlipidemia, n(%)  ALT, U/L  AST, U/L  Creatine, umol/L  BUN, mmol/L  NT-proBNP,ng/L  LDL-C,mmol/L  HDL-C,mmol/L  Triglyceride,mmol/L  ACEI/ARB/ARNI, n(%)  β-blocker, n(%)  CCB, n(%)  Diuretic, n(%)  Antiplatelet, n(%)  1-month mortality, n(%)  1-year mortality, n(%) | 66.5(58.0,73.0)  65(20.3)  212(66.2)  103(32.2)  10(3.1)  31.9(20.1,54.3)  81.4(36.1,183.8)  77.4(64.6,92.2)  6.4(5.1,7.4)  981.9(376.3,2562.7)  2.9(2.3,3.5)  1.0(0.9,1.2)  1.4(1.1,1.9)  5(1.6)  218(68.1)  25(7.8)  122(38.1)  318(99.4)  8(2.5%)  10(3.2%) | 66.0(58.0,73.0)  61(19.6)  206(66.0)  99(31.7)  9(2.9)  31.85(20.1,54.1)  80.7(36.1,178.8)  77.4(64.6,92.2)  6.4(5.1,7.4)  951.7(368.9,2444.2)  2.9(2.3,3.5)  1.0(0.9,1.2)  1.4(1.1,1.9)  5(1.6)  211(67.6)  25(8.0)  115(36.9)  310(99.4)  0  2(0.6) | 76.5(70.8,82.8)  4(50.0)  6(75.0)  4(50.0)  1(12.5)  31.8(16.5,)  111.6(70.2,312.3)  79.4(60.8,88.1)  7.6(5.1,11.3)  7187.7(5488.9,9618.4)  2.5(2.3,2.6)  1.1(1.0,1.3)  1.3(1.1,1.4)  0  7(87.5)  0  7(87.5)  8(100)  8(100)  8(100) | 0.003  0.095  0.880  0.478  0.607  0.001  0.034  0.633  0.003  0.001  0.266  0.380  0.597  1.000  0.420  0.868  0.011  1.000  0.001  0.001 |

Continuous variables are expressed a median (25th, 75th); Categorical variables are expressed as frequencies (percentages). PCI: Percutaneous coronary intervention; ALT: alanine aminotransferase; AST: aspartate aminotransferase; BUN: blood urea nitrogen; NT-proBNP: N-terminal pro brain natriuretic peptide; LDL-C: Low density lipoprotein cholesterol; HDL-C: High density lipoprotein cholesterol; ACEI: angiotensin-converting enzyme inhibitor; ARB: Angiotensin receptor antagonists; ARNI: Angiotensin Receptor-Neprilysin Inhibitor; CCB: calcium channel blockers.

**Supplementary Table 5.** Univariable and multivariable Cox regression analysis of the prognostic factors for one-month mortality.

| Variable | Univariate analysis | |  | | Multivariate analysis | |
| --- | --- | --- | --- | --- | --- | --- |
|  | HR (95% CI) | *P*-value |  |  | HR (95% CI) | *P*-value |
| Age  Male  PCI  Hypertension  Diabetes  Hyperlipidemia  ALT  AST  Creatine  BUN  NT-proBNP  Triglyceride  Hemoglobin  Platelet  CCB  ACEI/ARB/ARNI  Antiplatelet  Diuretic  β-blocker | 1.086(1.072-1.099)  0.515(0.390-0.681)  0.042(0.010-0.168)  1.805(1.374-2.372)  1.550(1.168-2.057)  1.458(1.108-1.916)  1.001(1.001-1.001)  1.001(1.001-1.001)  1.006(1.005-1;007)  1.124(1.108-1.140)  1.000(1.000-1.000)  0.796(0.675-0.939)  0.983(0.977-0.989)  0.995(0.993-0.998)  0.471(0.233-0.957)  0.198(0.147-0.267)  0.391(0.280-0.546)  1.116(0.844-1.475)  0.301(0.228-0.398) | <0.001  <0.001  <0.001  0.002  0.002  0.007  <0.001  <0.001  <0.001  <0.001  <0.001  0.007  <0.001  <0.001  0.037  <0.001  <0.001  0.442  <0.001 | |  | 1.051(1.028-1.073)  1.193(0.739-1.926)  0.071(0.010-0.512）  1.258(0.802-1.974)  1.710(1.136-2.574)  1.237(0.793-1.929)  1.001(1.001-1.001)  1.000(0.999-1.001)  0.996(0.992-1.001)  1.110(1.050-1.174)  1.000(1.000-1.000)  0.922(0.716-1.187)  0.999(0.988-1.009)  1.000(0.997-1.003)  0.628(0.227-1.737)  0.351(0.213-0.578)  1.384(0.810-2.368)  0.596(0.366-0.971) | <0.001  0.471  0.009  0.317  0.010  0.348  0.008  0.191  0.111  0.003  0.006  0.527  0.807  0.969  0.370  <0.001  0.235  0.038 |

PCI: Percutaneous coronary intervention; ALT: alanine aminotransferase; AST: aspartate aminotransferase; BUN: blood urea nitrogen; NT-proBNP: N-terminal pro brain natriuretic peptide; ACEI: angiotensin-converting enzyme inhibitor; ARB: Angiotensin receptor antagonists; ARNI: Angiotensin Receptor-Neprilysin Inhibitor; CCB: calcium channel blockers.

**Supplementary Table 6.** Univariable Cox analysis regarding the BAB index and the endpoint.

|  | 1-month mortality | *P* value | 1-year mortality | *P* value |
| --- | --- | --- | --- | --- |
| Training cohort  Internal validation cohort  External validation cohort | 2.951(2.645-3.294)  2.601(2.185-3.097)  3.271(1.595-6.708) | <0.001  <0.001  <0.001 | 2.756(2.513-3.022)  2.570(2.232-2.958)  3.483(1.828-6.637) | <0.001  <0.001  <0.001 |

**Supplementary Table 7.** Baseline characteristics of STEMI patients stratified based on the tertiles of the BAB index.

| Variable | Tertile 1 (n = 2641) | Tertile 2 (n = 2720) | Tertile 3 (n = 2641) | *P* |
| --- | --- | --- | --- | --- |
| Age, y  Male, n (%)  Hypertension, n (%)  Diabetes, n (%)  HFRS  CCI  KILLIP, n(%)  Ⅰ  Ⅱ  Ⅲ  Ⅳ  PCI, n (%)  ALT, U/L  AST, U/L  Creatine, umol/L  BUN,mmol/L  NT-proBNP, ng/L  LDL-C, mmol/L  HDL-C, mmol/L  Triglyceride, mmol/L  Hemoglobin, g/L  Platelet,10^9/L  ACEI/ARB/ARNI, n(%)  β-blocker, n (%)  CCB, n (%)  Diuretic, n (%)  Antiplatelet, n (%)  1-month mortality, n (%)  1-year mortality, n (%) | 59.00 (51.00,66.00)  2201 (83.34)  886 (33.55)  630 (23.85)  2.90(2.90,4.50)  0(0,1.00)  2159 (81.75)  400 (15.15)  42 (1.59)  40 (1.51)  1643 (62.21)  24.00 (16.00,36.00)  60.00 (29.00,128.00)  69.80 (60.40,80.00)  5.10 (4.20,6.06)  106.80 (53.96,209.80)  3.10 (2.56,3.65)  1.03 (0.89,1.20)  1.57 (1.10,2.36)  143.00 (132.00,153.00)  224.50 (191.00,267.00)  1785 (67.59)  1732 (65.58)  220 (8.33)  509 (19.27)  2421 (91.67)  24 (0.91)  32 (1.21) | 62.00 (54.00,70.00)  2030 (74.63)  924 (33.97)  732 (26.91)  2.90(2.90,5.20)  0(0,1.00)  1885 (69.30)  666 (24.49)  92 (3.38)  77 (2.83)  1654 (60.81)  32.60 (22.00,49.75)  107.00 (47.50,209.00)  68.60 (58.50,80.60)  5.18 (4.20,6.20)  645.00 (391.00,1047.75)  3.08 (2.56,3.67)  1.05 (0.91,1.25)  1.45 (1.06,2.08)  138.00 (127.00,149.00)  220.00 (184.00,262.00)  1937 (71.21)  1906 (70.07)  222 (8.16)  943 (34.67)  2460 (90.44)  47 (1.73)  86 (3.16) | 68.00 (60.00,77.00)  1769 (66.98)  980 (37.11)  845 (32.00)  4.40(2.90,6.70)  0(0,1.00)  1026 (38.85)  984 (37.26)  348 (13.18)  283 (10.72)  1178 (44.60)  46.00 (28.00,76.00)  156.90 (64.00,329.50)  79.90 (65.30,99.87)  6.50 (5.20,8.50)  2853.00 (1556.00,5852.00)  2.96 (2.42,3.61)  1.09 (0.92,1.30)  1.31 (0.97,1.82)  133.00 (120.00,146.00)  214.00 (174.00,263.00)  1628 (61.64)  1746 (66.11)  164 (6.21)  1573 (59.56)  2308 (87.39)  219 (8.29)  338 (12.80) | <.001  <.001  0.012  <.001  <.001  <.001  <.001  <.001  <.001  <.001  <.001  <.001  <.001  <.001  <.001  <.001  <.001  <0.01  <.001  <.001  0.005  <.001  <.001  <.001  <.001 |

Continuous variables are expressed a median (25th, 75th); Categorical variables are expressed as frequencies (percentages). STEMI: ST-segment elevated myocardial infarction; PCI: Percutaneous coronary intervention; ALT: alanine aminotransferase; AST: aspartate aminotransferase; BUN: blood urea nitrogen; NT-proBNP: N-terminal pro brain natriuretic peptide; LDL-C: Low density lipoprotein cholesterol; HDL-C: High density lipoprotein cholesterol; ACEI: angiotensin-converting enzyme inhibitor; ARB: Angiotensin receptor antagonists; ARNI: Angiotensin Receptor-Neprilysin Inhibitor; CCB: calcium channel blockers.

**Supplementary Table 8**. Baseline characteristics of patients for different culprit vessels.

| Variables | LAD (n = 462) | LCX (n = 129) | RCA (n = 301) | *P* |
| --- | --- | --- | --- | --- |
| Age, y  Platelet, 10^9/L  Hemoglobin, g/L  Triglyceride, mmol/L  LDL-C, mmol/L  HDL-C, mmol/L  ALT, U/L  AST, U/L  BUN, mmol/L  Male, n(%)  KILLIP, n(%)  Ⅰ  Ⅱ  Ⅲ  Ⅳ  PCI, n(%)  Diabetes, n(%)  Hypertension, n(%)  Hyperlipidemia, n(%)  1-month mortality, n(%)  1-year mortality, n(%)  CCB, n(%)  β-blocker, n(%)  Tatin, n(%)  Diuretic, n(%)  Antiplatelet, n(%)  ACEI/ARB/ARNI, n(%) | 63.00 (56.00,71.00)  217.00 (180.00,255.00)  136.00 (126.00,149.00)  4.58 (3.95,5.24)  3.01 (2.48,3.59)  1.09 (0.89,1.27)  33.00 (20.00,55.00)  120.00 (48.00,250.15)  5.61 (4.50,6.90)  355 (76.84)  309 (66.88)  108 (23.38)  20 (4.33)  25 (5.41)  261 (56.49)  123 (26.62)  173 (37.45)  116 (25.11)  19 (4.11)  27 (5.84)  35 (7.58)  300 (64.94)  417 (90.26)  168 (36.36)  419 (90.69)  307 (66.45) | 62.00 (54.00,72.00)  222.50 (171.00,261.00)  139.00 (128.00,148.00)  4.70 (4.01,5.28)  2.99 (2.53,3.71)  1.06 (0.91,1.22)  34.20 (21.00,58.00)  105.00 (49.75,251.00)  5.30 (4.30,7.10)  96 (74.42)  82 (63.57)  33 (25.58)  7 (5.43)  7 (5.43)  69 (53.49)  35 (27.13)  39 (30.23)  32 (24.81)  6 (4.65)  10 (7.75)  10 (7.75)  90 (69.77)  117 (90.70)  47 (36.43)  119 (92.25)  88 (68.22) | 63.00 (55.00,72.00)  228.00 (184.00,269.00)  139.00 (126.00,151.00)  4.67 (4.14,5.33)  3.13 (2.59,3.71)  1.03 (0.89,1.22)  29.00 (19.00,46.00)  89.30 (40.50,157.00)  5.33 (4.24,6.80)  225 (74.75)  194 (64.45)  78 (25.91)  17 (5.65)  12 (3.99)  164 (54.49)  97 (32.23)  97 (32.23)  82 (27.24)  10 (3.32)  16 (5.32)  26 (8.64)  188 (62.46)  263 (87.38)  118 (39.20)  270 (89.70)  198 (65.78) | 0.720  0.073  0.252  0.168  0.181  0.621  0.010  0.015  0.188  0.746  0.886  0.773  0.228  0.175  0.775  0.774  0.614  0.865  0.346  0.390  0.712  0.705  0.887 |

Continuous variables are expressed a median (25th, 75th); Categorical variables are expressed as frequencies (percentages). LAD: left anterior descending artery; LCX: left circumflex artery; RCA: right coronary artery; PCI: Percutaneous coronary intervention; ALT: alanine aminotransferase; AST: aspartate aminotransferase; BUN: blood urea nitrogen; NT-proBNP: N-terminal pro brain natriuretic peptide; LDL-C: Low density lipoprotein cholesterol; HDL-C: High density lipoprotein cholesterol; ACEI: angiotensin-converting enzyme inhibitor; ARB: Angiotensin receptor antagonists; ARNI: Angiotensin Receptor-Neprilysin Inhibitor; CCB: calcium channel blockers.

**Supplementary Table 9**. Diagnostic results of collinearity for predictive features of one-month mortality risk

| **Variables** | **VIF** | **Variables** | **VIF** |
| --- | --- | --- | --- |
| TC | 9.032 | Hepatic insufficiency | 1.421 |
| Statins | 7.276 | KILLIP | 1.384 |
| Antiplatelet | 7.201 | Diuretic | 1.372 |
| LDL-C | 7.159 | AST | 1.347 |
| BAB | 3.491 | Hypertension | 1.322 |
| Cardiac insufficiency | 2.236 | Gender | 1.322 |
| Cr | 2.188 | Diabetes | 1.265 |
| TG | 2.18 | HFRS | 1.203 |
| Renal insufficiency | 1.757 | TP | 1.193 |
| HDL-C | 1.691 | PCI | 1.118 |
| Age | 1.614 | PLT | 1.105 |
| CCI | 1.558 | AF | 1.034 |
| β-blocker | 1.518 | III°AVB | 1.008 |
| ACEI/ARB/ARNI | 1.503 | VF | 1.007 |
| UA | 1.43 |  |  |

TC: total cholesterol; LDL-C: Low density lipoprotein cholesterol; Cr: creatine; TG: Triglyceride; HDL-C: High density lipoprotein cholesterol; CCI: Charlson Comorbidity Index; HFRS: Hospital Frailty Risk Score; ACEI: angiotensin-converting enzyme inhibitor; ARB: Angiotensin receptor antagonists; ARNI: Angiotensin Receptor-Neprilysin Inhibitor; UA: urea acid; AST: aspartate aminotransferase; TP: total protein; PCI: Percutaneous coronary intervention; PLT: platelet; AF: atrial fibrillation; AVB: atrioventricular block; VF: ventricular fibrillation.

**Supplementary Table 10.** Diagnostic results of collinearity for predictive features of one-year mortality risk

| **Variables** | **VIF** | **Variables** | **VIF** |
| --- | --- | --- | --- |
| TC | 9.138 | β-blocker | 1.519 |
| Statin | 7.285 | Hypertension | 1.491 |
| LDL-C | 7.241 | CCI | 1.437 |
| Antiplatelet | 7.211 | UA | 1.437 |
| BAB | 3.532 | Hepatic insufficiency | 1.426 |
| ALB | 2.493 | KILLIP | 1.391 |
| Cardiac insufficiency | 2.238 | Hyperlipidemia | 1.386 |
| Cr | 2.214 | Diuretic | 1.371 |
| TG | 2.191 | AST | 1.35 |
| TP | 2.094 | HFRS | 1.207 |
| Renal insufficiency | 1.759 | PCI | 1.128 |
| HDL-C | 1.748 | PLT | 1.107 |
| Age | 1.73 | CCB | 1.055 |
| Hb | 1.665 | AF | 1.035 |
| Gender | 1.576 | III°AVB | 1.008 |
| ACEI/ARB/ARNI | 1.525 | VF | 1.007 |

TC: total cholesterol; LDL-C: Low density lipoprotein cholesterol; ALB: albumin; Hb: hemoglobin; Cr: creatine; TG: Triglyceride; HDL-C: High density lipoprotein cholesterol; CCI: Charlson Comorbidity Index; HFRS: Hospital Frailty Risk Score; ACEI: angiotensin-converting enzyme inhibitor; ARB: Angiotensin receptor antagonists; ARNI: Angiotensin Receptor-Neprilysin Inhibitor; UA: urea acid; AST: aspartate aminotransferase; TP: total protein; PCI: Percutaneous coronary intervention; PLT: platelet; AF: atrial fibrillation; AVB: atrioventricular block; VF: ventricular fibrillation; CCB: calcium channel blockers.

**Supplementary Table 11.** Comparison of the predictive efficacy of different prediction models for the 1-month mortality risk of patients in the test set

| **Algorithms** | **AUC(95%CI)** | **Accuracy (95%CI)** | **Sensitivity(95%CI)** | **Specificity(95%CI)** | **F1 Score(95%CI)** |
| --- | --- | --- | --- | --- | --- |
| XGBoost | 0.873 (0.824-0.923) | 0.846(0.824-0.868) | 0.8(0.765-0.835) | 0.848(0.824-0.872) | 0.276(0.254-0.299) |
| Logistic | 0.817 (0.765-0.869) | 0.711(0.692-0.731) | 0.776(0.748-0.804) | 0.709(0.688-0.730) | 0.163(0.156-0.171) |
| LightGBM | 0.808 (0.746-0.871) | 0.87(0.842-0.897) | 0.666(0.645-0.686) | 0.877(0.849-0.906) | 0.278(0.230-0.325) |
| AdaBoost | 0.907 (0.875-0.940) | 0.803(0.787-0.819) | 0.862(0.836-0.888) | 0.801(0.783-0.819) | 0.242(0.230-0.253) |
| GBDT | 0.773 (0.706-0.840) | 0.888(0.874-0.903) | 0.583(0.515-0.651) | 0.9(0.886-0.913) | 0.277(0.232-0.321) |

AUC: area under the curve; CI: confidence interval.

**Supplementary Table 12.** Comparison of the predictive efficacy of different prediction models for the 1-year mortality risk of patients in the test set

| **Algorithms** | **AUC(95%CI)** | **Accuracy (95%CI)** | **Sensitivity(95%CI)** | **Specificity(95%CI)** | **F1 Score(95%CI)** |
| --- | --- | --- | --- | --- | --- |
| XGBoost | 0.871 (0.836-0.905) | 0.839(0.813-0.866) | 0.73(0.653-0.808) | 0.846(0.814-0.878) | 0.343(0.325-0.361) |
| Logistic | 0.777 (0.732-0.823) | 0.644(0.616-0.671) | 0.783(0.722-0.844) | 0.635(0.606-0.665) | 0.201(0.185-0.217) |
| LightGBM | 0.776 (0.728-0.823) | 0.847(0.808-0.885) | 0.629(0.520-0.738) | 0.86(0.813-0.907) | 0.324(0.297-0.350) |
| AdaBoost | 0.885 (0.856-0.913) | 0.787(0.767-0.807) | 0.82(0.750-0.890) | 0.785(0.761-0.809) | 0.306(0.289-0.322) |
| GBDT | 0.763 (0.715-0.812) | 0.846(0.788-0.904) | 0.59(0.388-0.792) | 0.861(0.788-0.935) | 0.304(0.260-0.347) |

AUC: area under the curve; CI: confidence interval.
